# Metabolic Responses to Low Temperature of Three Peach Fruit Cultivars Differently Sensitive to Cold Storage

**DOI:** 10.3389/fpls.2018.00706

**Published:** 2018-05-28

**Authors:** Stefano Brizzolara, Maarten Hertog, Roberta Tosetti, Bart Nicolai, Pietro Tonutti

**Affiliations:** ^1^Istituto di Scienze della Vita, Scuola Superiore Sant’Anna, Pisa, Italy; ^2^Division of Mechatronics, Biostatistics and Sensors (MeBioS), Department of Biosystems (BIOSYST), KU Leuven, Leuven, Belgium; ^3^Flanders Centre of Postharvest Technology (VCBT), Leuven, Belgium

**Keywords:** chilling injury, cold storage, correlation analysis, mesocarp, metabolomics, *Prunus persica*, shelf-life, volatile organic compounds (VOCs)

## Abstract

Refrigerated storage is widely applied in order to maintain peach quality but it can also induce chilling injuries (CIs) such as flesh browning and bleeding, and mealiness. Peach fruit from three cultivars (‘Red Haven’, RH, ‘Regina di Londa’, RL, and ‘Flaminia’, FL) were stored for 4 weeks under low temperatures (0.5 and 5.5°C). GC-MS was employed to study changes in both metabolome and volatilome induced by cold storage in the mesocarp. CIs were assessed both at the end of each week of storage and after subsequent shelf-life (SL) at 20°C. Flesh browning and mealiness appeared to be more related to 5.5°C storage, while flesh bleeding revealed high incidence following 0.5°C storage. Compared to RL and FL, RH showed a marked lower incidence of CIs. Multivariate statistical analyses indicate that RH peaches indeed differ from RL and FL in particular when considering data from samples collected at the end of the cold storage. Common and divergent responses have been identified in terms of metabolic responses to the applied low temperatures. In all three cultivars raffinose, glucose-6P, fucose, xylose, sorbitol, GABA, epicatechin, catechin, and putrescine markedly increased during cold storage, while citramalic, glucuronic, mucic and shikimic acids decreased. Among volatile organic compounds (VOCs), aldehydes and alcohols generally accumulated more under low temperature conditions while esters and lactones evolved during subsequent SL. The main cultivar differences developed after cold storage during SL although some common responses (e.g., an increased production of ethyl acetate) were observed. The lower levels of flesh browning and bleeding displayed by RH peaches were related to compounds with antioxidant activity, or acting as osmotic protectants and membrane stabilizer. Indeed, RH showed higher levels of amino acids and urea, together with a marked increase in putrescine, sorbitol, maltitol, myoinositol and sucrose detected during storage and SL.

## Introduction

When kept at room temperature peach fruit undergo a rapid postharvest loss of firmness, weight loss and decay (Ramina et al., 2008). Therefore peaches are commonly stored under low temperatures, with benefits for the commercial life that is prolonged up to 2–4 weeks. One of the problems caused in peaches by cold storage is the onset of chilling injuries (CIs), manifested as loss of flavor and the ability to ripen, increased incidence of decay, internal browning, flesh breakdown, lack of juiciness (mealiness/woolliness), and finally reddish flesh discolouration (reddening/bleeding) (Crisosto and Mitchell, 2002; Lurie and Crisosto, 2005). The internal structure of the mesocarp and the phenotype (e.g., melting/non-melting, freestone/clingstone) affect the incidence and visual appearance of the disorders.

The CI symptoms typically develop after cold storage when fruit are moved to SL conditions (Crisosto et al., 1995). Between 2.2 and 7.6°C (the so-called “killing zone”) these symptoms develop faster and more severely than at 0°C or below but above the freezing point (Crisosto and Valero, 2008). The onset of peach fruit disorders under the “killing zone” temperature appears to be linked with the low levels of ethylene biosynthesis only partly inducing ripening-related genes (Fernández-Trujillo et al., 1998; Walsh et al., 2001; Zhou et al., 2001; Pons et al., 2015; Wang et al., 2017). However, the specific temperature requirement to maintain fruit quality may vary in relation to the genetic background resulting in different metabolic reactions that impact on both the organoleptic properties (aroma compounds, primary and secondary metabolites) (Oms-Oliu et al., 2013; Johanningsmeier et al., 2016) and the incidence of the storage physiological disorders (Dagar et al., 2013; Pons et al., 2015).

The genetic background plays a main role in the modulation of peach fruit responses to low temperatures and in the onset of CIs. Bustamante et al. (2016), working on six different peach melting and non-melting varieties showing variable responses to chilling, demonstrated that the content of specific metabolites (e.g., raffinose, xylose) correlate to the degree of mealiness and concluded that the differential metabolic re-arrangements induced by cold storage are somehow linked to the different CI resistance/susceptibility. Based on that, the same authors identified possible molecular markers of cold-related disorders. A metabolic re-arrangement is associated with the induction of acquired CI tolerance (priming) in peaches undergoing heat treatment before cold storage (Lauxmann et al., 2014). Indeed, the onset of CIs is strictly dependent on the temperature regimes that induce selective metabolic reactions resulting in fruit structure and appearance alterations. Wang et al. (2013) observed different levels of sucrose, glucose, and fructose in peaches stored at 0 and 5°C, and this has been related to membrane stability and sensitivity to CIs.

The different low temperature regimes also affect one of the most important organoleptic parameters of peaches represented by the volatile organic compounds (VOCs). It is well known that in ripening peaches, several dozens of VOCs, belonging to aldehydes, alcohols, alkanes, ketones, lactones, terpenes, and esters are synthesized (Aubert and Milhet, 2007; Yang et al., 2009), making fruit attractive for consumption. Some papers report the effects of cold storage on volatile profile and pathways in peach fruit (Zhang et al., 2011, 2012; Cano-Salazar et al., 2013). In these works, several volatile chemical classes (mainly lactones, esters and aldehydes), and some specific compounds, vary significantly during ripening after cold storage and may influence consumer acceptance.

Most of the papers mentioned above focus either on VOCs or metabolic profiling. In order to better elucidate the complex metabolic responses of peach fruit to cold storage conditions we performed integrated and correlation analyses of the metabolome and volatilome of mesocarp samples from three melting peach cultivars stored under two low temperature regimes followed by shelf-life (SL) at room temperature.

## Materials and Methods

### Plant Material and Sampling Procedure

Peach (*Prunus persica* L. *Batsch)* fruit belonging to cultivars ‘Red Haven’ (RH, yellow fleshed) ‘Flaminia’ (FL, yellow-fleshed, +35 days from RH harvest), ‘Regina di Londa’ (RL, white-fleshed, +55 days from RH harvest) were harvested at flesh firmness values of 37, 42, and 56 N for RH, FL, and RL, respectively, from a commercial orchard located at Casciana Terme (Pisa, Tuscany, Italy). These cvs have been selected based on previous results (Brizzolara and Tonutti, unpublished) demonstrating different postharvest behavior and CI incidence following low temperature storage. Homogeneous fruit in terms of size and peel color were further selected using NIR technology (NIR-Case, SACMI, Italy). The NIR-Case has been calibrated for fruit firmness (manual penetrometer), total soluble solids (TSS) (optical refractometer) and peel color (arbitrary scale) on more than 300 peaches per cultivar. Immediately after transfer to the lab, fruit were incubated in cold chambers at two low temperature conditions (0.5 and 5.5°C) and at 20°C (±0.5°C), the latter samples representing the reference (control) of post-harvest ripening. Relative humidity under cold storage was kept at 85%, and monitored using a TGU-4500 (Tinytag Ultra 2, Gemini, United Kingdom) data logger. Peaches were sampled at harvest (T0) and after 1, 2, 3, and 4 weeks of cold storage, whereas for the control at 20°C the 4th week sampling was not performed due to over-ripening and decay. Considering that CI symptoms appear after fruit removal from cold storage, fruit were additionally kept for 3 days at room temperature to evaluate the post-storage behavior and the shelf-life (SL). For metabolomics analyses three fruit were processed for each sample and a random pool of sound or corrupted (in CI- affected fruit) mesocarp tissue immediately frozen in liquid nitrogen and stored at -80°C. For VOC analysis, mesocarp samples of the same three fruit were crushed in a 0.5 M NaCl solution using a T25 Ultra-Turrax^®^ (IKA, Germany) and subsequently frozen in liquid nitrogen. The NaCl concentration has been choosen based on preliminary trials and previous experiments on peach fruit VOC analysis. Firmness (expressed in N) was measured using a manual penetrometer equipped with 8 mm tip while total soluble solids (TSS, %) content was determined using an optical refractometer. These analyses were performed on nine individual fruit.

### Evaluation of CI Symptoms

Fruit were evaluated for different CI symptoms, such as flesh browning, flesh bleeding, and mealiness. Considering flesh browning and bleeding, nine fruit were evaluated for each sampling time. Fruit were cut in two halves; for each part, both brown and red areas were assessed separately with the open source imaging software ‘ImageJ,’ resulting in 18 measurements for each treatment at a specific sampling time. Half fruit was considered as representing 50% of the whole fruit. If the cutting surface of one half was completely red or brown it was counted as 50% of CI incidence for that specific fruit. The incidence (%) of each treatment was calculated as the average of the nine tested peaches. Peach juiciness has been evaluated by processing with an electric juice extractor (Moulinex Juice Extractor JU350B27) 50 g of pooled tissue from three peaches (in triplicates, nine fruit in total). Values were expressed as percentage of extracted juice normalized on control levels.

### Derivatization of Methanolic Extracts

The derivatization method used by Brizzolara et al. (2017) has been applied with several changes. The frozen pulp was grinded using a mixer mill (Retsch, MM 200) shaking at 20 Hz for 1 min. 200 mg of frozen powder was weighed into a 2 mL microcentrifuge eppendorf tube with 1 mL of ice-cold methanol and incubated using a thermomixer at 70°C for 15 min and 1,400 rpm. After centrifugation (23,000 *g*, 20 min, 4°C), 100 μL of the supernatant was transferred to a new 1.5 ml microcentrifuge tube and 50 μL of the internal standard mix, containing phenyl-β-D-glucopyranoside (3 μg/μl methanol) and 3-4 hydroxy-phenyl propionic acid (0.1 μg/μl methanol), was added to the supernatant. The samples were immediately dried under a stream of nitrogen gas for 50 min at 50°C. The dried samples were redissolved in 50 μL of MOX (a solution of 20 mg methoxyamine hydrochloride in 1 mL of pyridine) and incubated at 30°C for 60 min while shaking. Finally, derivatization of the mixture was achieved through incubation with 120 μL of BSTFA (*N,O*-bis(trimethylsilyl)trifluoroacetamide) at 45°C for 120 min while shaking. For all samples, 1 μL of the derivatized extract was injected on the GC column of an Agilent GC-MS system [GC 7890 with a 5975 single quadrupole MS with electron impact ionization source (Agilent Technologies, Palo Alto, CA, United States)]. Each sample was analyzed twice; a split (1:150) method was used for the abundant compounds whereas a splitless method was employed for the less abundant compounds. A HP-5MS capillary GC-MS column of 30 m length, 0.25 mm internal diameter and 0.25 μm film thickness (Supelco, Bellefonte, CA, United States) was employed. The injector and interface temperatures were 220 and 280°C, respectively. Helium was used as a carrier gas with an average flow rate of 1 ml min^-1^. The GC temperature program started isothermal at 50°C for 1 min (acids method) or at 120°C for 1 min (sugars method), and was then ramped at a rate of 10°C/min to 310°C where it was kept for 13 min (acids method) or to 300°C where it was kept for 6 min (sugars method). The total run time for the acids method was 40 min, and that for the sugars method was 25 min. Mass spectra in the 50–600 m/z range were recorded at a scanning speed of 2.66 scan cycles per second. The MS ion source and quadrupole temperatures were 230 and 150°C, respectively.

### HS-SPME-GC-MS Analysis

For aroma volatile compound analysis a method previously adopted by Brizzolara et al. (2017) was used. Stored samples were thawed in a 15°C water bath (SWB20, Haake GmbH, Germany) and 10 g were transferred into a 20 mL vial. Analyses have been performed using an Agilent Technologies (6890N, United States) gas chromatograph equipped with an auto-sampler (MPS2, Gerstel Multipurpose sampler, Germany). The samples were incubated at 40°C for 2 h. Subsequently, the volatile compounds were sampled for 45 min by means of a solid phase micro extraction (SPME) fiber (Supelco Inc., Bellefonte, PA, United States), with a polydimethylsiloxane/divinylbenzene (PDMS/DVB, 1 cm long, 65 μm thickness, 0.357 μL volume) sorptive coating. The fiber was desorbed into the split/splitless liner of the GC for 5 min in splitless mode, setting at 250°C the injector temperature. Volatiles were separated on a 30 m × 0.25 mm i.d. capillary column (HP-5MS, 5% phenyl methyl siloxane) having a film thickness of 0.25 μm. Helium was the carrier gas with a flow rate of 1.2 mL min^-1^. The GC oven heating started at 40°C and was increased to 250°C at a rate of 5°C min^-1^ with a total analysis time of 32.5 min. Each sample was analyzed in quadruplicate. For the identification of the compounds a mass spectrometer (5973 Network Mass Selective Detector, Agilent Technologies) coupled to the GC was used.

### Compound Identification

Each chromatogram was deconvoluted using the automated mass spectral deconvolution and identification system (AMDIS, National Institute of Standards and Technology, Gaithersburg, MD, United States).

Considering the analysis of the derivatized methanolic extracts, compound identification was carried out by comparing the peak retention indices (RI) and mass spectra against a home-built library of commercial standards. Standards were purchased from Sigma-Aldrich-Fluka (Diegem, Belgium) (pyruvic acid, benzoic acid, phosphoric acid, glyceric acid, glutamic acid, alanine, valine, phenylalanine, asparagine, serine, threonine, sucrose, galactose, glucose, sorbitol, urea), Acros Organics (Geel, Belgium) (lactic acid, succinic acid, fumaric acid, quinic acid, aspartic acid, mannose, fructose, cellobiose, erythritol, ribitol), Merck Chemicals (Overijse, Belgium) (malic acid, xylose) and VWR (BDH Prolabo, Leuven, Belgium) (mannitol). The quantification of the compounds was performed using the MSD ChemStation software (Agilent Technologies, Palo Alto, CA, United States) in order to collect the peak areas. Raw peak area data were normalized using the actual peak area of the internal standard (phenyl β-D-glucopyranoside).

Regarding the HS-SPME-GC-MS chromatograms, each peak was identified by comparing the experimental spectra with those of the National Institute for Standards and Technology (NIST98, Version 2.0, United States) data bank considering only results with 85%, or more, of matching. Peak retention indices (RI) have been used to optimize data bank screening. Raw peak area data were normalized based on the sum of the areas of all the identified peaks for each sample.

### Statistical Analyses

Principal component analysis (PCA) and partial least squares discriminant analysis (PLS-DA) were performed on the normalized data of the experiment, using a dataset containing results from both GC-MS and HS-SPME-GC-MS analyses, using the JMP software (JMP^®^, Version *13* SAS Institute Inc., Cary, NC, United States, 1989–2007). PLS analyses have been performed using the metabolites and the measured quality parameters as predictor variables while employing cumulative temperature [calculated using the following formula: “Time^∗^Temperature” = storage time (days) ^∗^ storage temperature (°C)] or cultivar (‘Flaminia,’ ‘Red Haven,’ and ‘Regina di Londa’) as response variables. All variables were mean centered and weighed by their standard deviation to assign them equal variance. Variable importance in projection (VIP) scores were employed to filter the PLS results selecting important features.

Data presented were analysed using one-way analysis of variance (ANOVA) and independent samples *t*-test statistical tools (*p* ≤ 0.05) in order to identify compounds significantly differing between the tested treatments. Correlation analyses between detected compounds based on Pearson correlation and hierarchical clustering analysis (HCA) have been performed employing Metaboanalyst online tool (Xia and Wishart, 2016). Specifications for each analysis are reported in the relative captions.

The metabolomic network has been constructed using the ExpressionCorrelation plug-in^1^ for Cytoscape software (Shannon et al., 2003). Pearson correlation coefficient cut-off values were set at -0.7 and 0.7, and the network has been visualized using the Cytoscape software v2.7.0^2^ .

## Results

### Chemico-Physical Parameters

**FIGURE 1:**
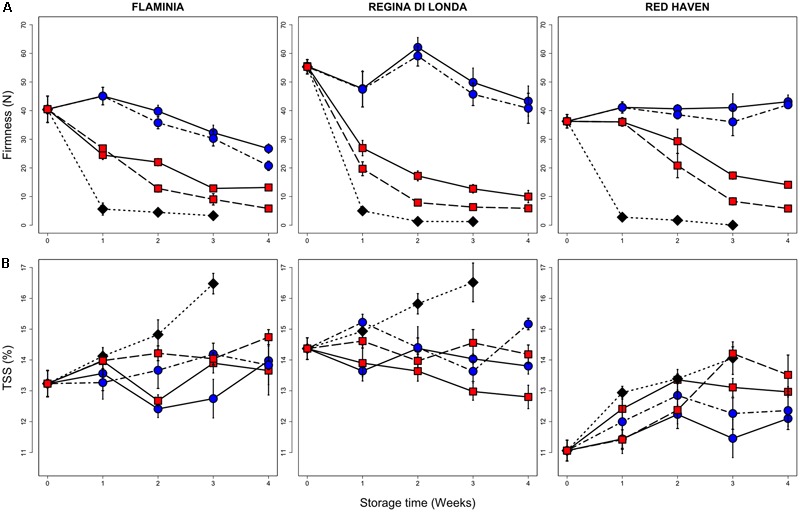
Flesh firmness **(A)** and TSS **(B)** measured in the three peach varieties. All measurements have been performed on fruit stored up to three (control, 20°C) or four (0.5 and 5.5°C) weeks and then kept for three additional days under shelf-life conditions (20°C, SL; dotted lines), and on peaches sampled immediately after cold storage (no SL; solid lines). Values are the mean of nine biological replicates and bars represent SE. Black diamonds, blue circles and red squares represent control fruit, 0.5 and 5.5°C stored peaches, respectively.

Cold storage conditions affected the quality parameters of all tested peach varieties (**Figure 1**). As expected, flesh firmness rapidly decreased in control samples (kept at 20°C) already after 1 week, reaching firmness values lower than 10 N (unmarketable fruit). Firmness loss was reduced in samples stored at 0.5°C in all varieties, whereas less pronounced effects were observed at 5.5°C, both under and after storage (SL), with RH showing a better firmness retention if compared to FL and RL (**Figure 1**). Total soluble solids (TSS), higher at harvest in FL and RL as compared to RH, showed an increasing trend in FL and RL control fruit while in refrigerated samples no marked changes were observed. RH samples kept at 0.5°C had the lowest TSS values after 3 and 4 weeks of storage (**Figure 1**).

### Incidence of CIs During and After Cold Storage

**FIGURE 2:**
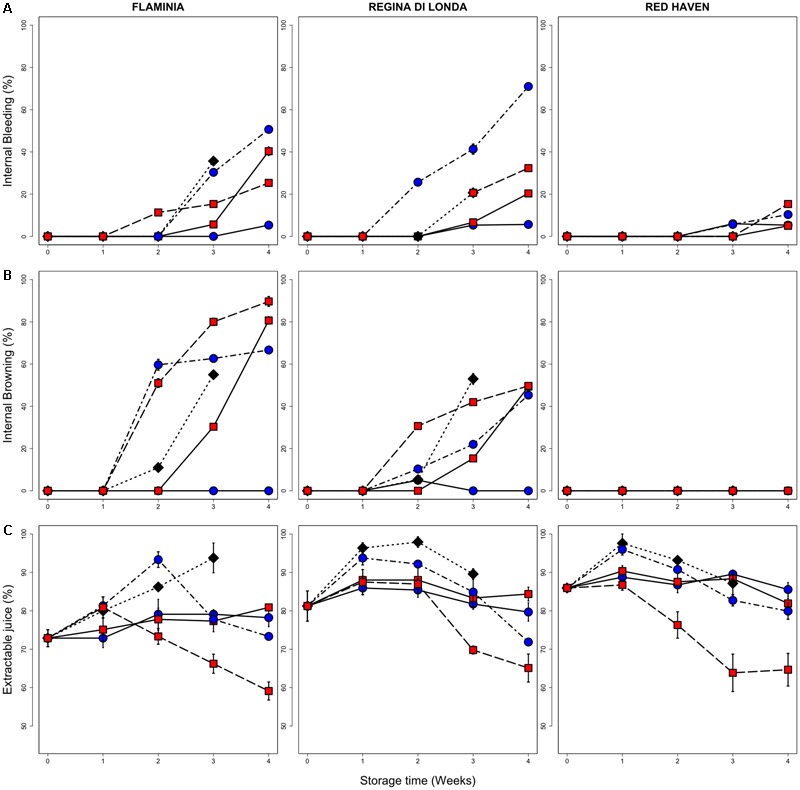
Incidence of CIs (flesh bleeding, **A**; flesh browning, **B**; and loss of juiceness, **C**) in the three peach fruit varieties. All measurements have been performed on fruit stored up to three (control, 20°C) or four (0.5 and 5.5°C) weeks and then kept for three additional days under shelf-life conditions (20°C, SL; dotted lines), and on peaches sampled immediately after cold storage (no SL; solid lines). Values are the mean of nine (browning and bleeding) and three (loss of juiciness) biological replicates and bars represent SE. Black diamonds, blue circles and red squares represent control fruit, 0.5 and 5.5°C stored peaches, respectively.

The evaluation of the incidence of flesh disorders reveals differences among varieties (**Figure 2**). While in RH a very limited incidence of flesh bleeding and no browning were observed, both RL and FL developed CI symptoms, in particular in samples evaluated after 3 days of SL, with some difference between 0.5 and 5.5°C. In RL and FL the different behavior between the two low temperature conditions is particularly evident when comparing fruit evaluated immediately after storage (no SL), with higher CI incidence detected in samples kept at 5.5°C (**Figure 2**, solid lines). In all cultivars, flesh bleeding appeared to be induced more by storage at 0.5°C while, though less markedly, browning seemed to be more induced by 5.5°C storage (**Figure 2**).

Considering the parameter of juiciness, an increasing trend was observed in control fruit, while, under cold storage at both 0.5 and 5.5°C, its level appeared quite stable in all the studied varieties. Peaches sampled after storage at 5.5°C (SL) showed the lowest extractable juice values, in particular in fruit stored for 3 and 4 weeks (**Figure 2**).

### The Effects of Cold Storage on Metabolic Composition

A total of 57 and 54 compounds were detected employing metabolomics and aroma profiling approach, respectively (Supplementary Tables S1, S2). All the compounds identified via derivatization GC-MS analysis were found in all cultivars, while for the aroma volatiles, next to the common VOCs, cultivar-specific aroma compounds produced by only one or two of the three varieties were detected.

**FIGURE 3:**
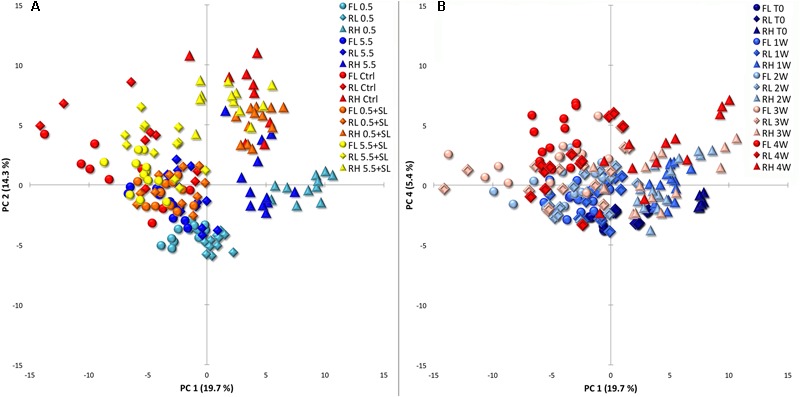
Principal component analysis (PCA) of cold storage and shelf-life (SL) data together pointing out the effects of storage conditions (**A**, PC1 vs. PC2) and storage time (**B**, PC1 vs. PC4). Circles, diamonds and triangles are used depicting ‘Flaminia’ (FL), ‘Regina di Londa’ (RL), and ‘Red Haven’ (RH) peaches, respectively, in both **(A,B)**. **(A)** Light blue, blue, red, orange, and yellow colors indicate 0.5°C, 5.5°C, control, 0.5°C + SL and 5.5°C + SL peaches, respectively, including in each cluster samples from 0 to 4 weeks of storage (3 weeks for control fruit). **(B)** Colors from dark blue to red indicate samples from 0 to 4 weeks of storage (3 weeks for control fruit) and each group includes samples from all the tested conditions.

The whole dataset (derivatization, HS-SPME and chemico-physical parameters) was investigated via multivariate statistical analysis and the three varieties collected during both cold storage and SL were considered together. A PCA revealed a total described variation of 34% considering overall PC 1 and PC 2 (**Figure 3A**). In this projection, RH peaches appear to be separated from FL and RL, which group together, but the different cold treatments are not clearly separated (**Figure 3A**). Cold stored samples in general sit in the lower quadrants while stored peaches after SL and control fruit sit overlapped in the upper part of the graph. In RH peaches this clustering is more evident with cold stored fruit almost separated from all the others. In all cultivars, samples stored at 0.5°C tend to cluster separate from the bulk produced by control and SL peaches (**Figure 3A**). Even though it only describes 5.4% of the overall variation, the fourth component indicates a time effect showing fruit at harvest sitting in the lower quadrants and most of peaches after 4 weeks of storage in the upper quadrants (**Figure 3B**).

### Genotype-Related Differences of Metabolic and Aroma Profiling

**FIGURE 4:**
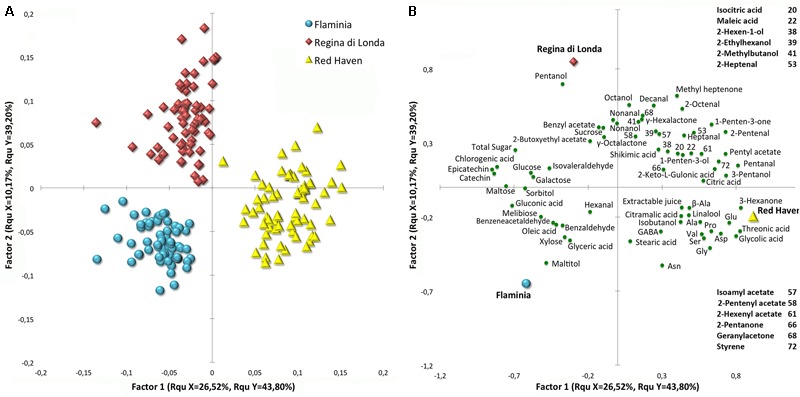
Score **(A)** and loading **(B)** plots of a PLS-DA analysis performed with the whole dataset, including cold storage and shelf-life experiments together. All the detected compounds were employed as predictor variables and cultivar as response variable. Important features have been filtered using VIP (Variable Importance in Projection) scores and are reported in the loading plot **(B)** as green circles. Blue circles, red diamonds, and yellow triangles represent ‘Flaminia,’ ‘Regina di Londa,’ and ‘Red Haven’ peaches, respectively.

When PLS analysis was carried out using cultivar as response variable and the identified metabolites or VOCs separately, as predictor variables, high percentages of explained variable were obtained (a total of about 75 and 77%), respectively, and samples from the three cultivars appear to be well separated (Supplementary Figure S1). A PLS analysis, using the whole set of metabolites and VOCs data as predictor variables, shows that the three cultivars are characterized by specific composition and metabolic responses during and after cold storage (**Figures 4A,B**). Variable importance in projection (VIP) scores have been employed to select important features contributing to the separation. Metabolite profiles markedly varied among varieties contributing to the specific flavor of each variety.

The most evident class of compounds contributing to cultivar clustering in the PLS analysis is amino acids, which is associated with RH peaches (**Figure 4B**). All the identified metabolites belonging to this chemical class group in the right lower quadrant of the graph, where RH samples are located. Furthermore, RH and RL samples appear to be generally richer in organic acids, alcohols and aldehydes, with RL cultivar showing higher levels of several compounds such as pentanol, octanol, nonanol, and 2-methylbutanol, whereas RH peaches reveal higher amounts of threonic and glycolic acids (**Figure 4B**). On the other hand, FL and RL cultivars appear to be more related to sugars and sugar alcohols in general, but also to flavonols, benzeneacetaldehyde, and benzaldehyde. Maltitol, sorbitol, maltose, melibiose, galactose, xylose, glyceric and gluconic acid are the compounds which contribute the most to FL peaches clustering.

### The Specific Effects of Low Temperatures and the Post-storage Shelf-Life

**FIGURE 5:**
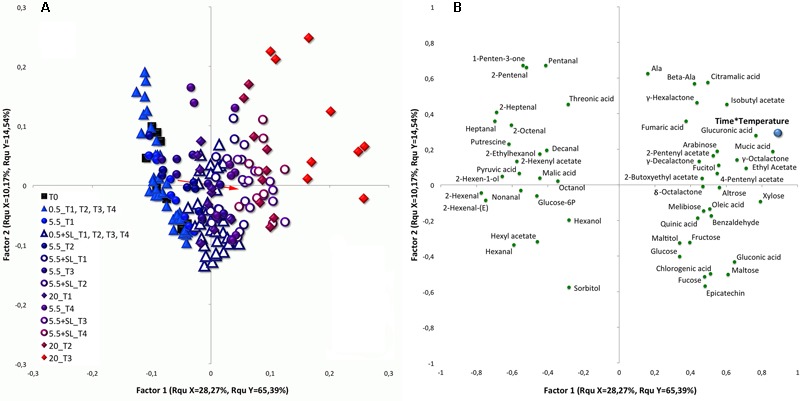
Score **(A)** and loading **(B)** plots of a PLS-DA analysis performed with the whole dataset, including cold storage and shelf-life (SL) experiments together. The identified compounds were used as predictor variables and cumulative temperature (Time^∗^Temperature, see section “Statistical Analyses” for additional information) was employed as response variable. Filled black squares were used depicting samples at harvest (T0). Filled and empty triangles represent peaches under and after cold storage at 0.5°C (up to 4 weeks), respectively (0.5°C; 0.5°C + SL). Filled and empty circles were used to color fruit under and after storage at 5.5°C (up to 4 weeks), respectively (5.5°C; 5.5°C + SL). Control fruit (20°C, up to 3 weeks) were drawn as filled diamond. Colors from light blue to intense red were used to depict samples (T0 excluded) depending on their specific “Time^∗^Temperature” value. Important features have been filtered using VIP (Variable importance in projection) scores and are reported in the loading plot **(B)** as green circles. The red arrow indicates the displacement relative to the overall variation existing between the samples under storage and during SL. The bottom tail of the arrow has been drawn using the average coordinates of samples at harvest and under cold storage (both under 0.5 and 5.5°C), whereas to set the head value the mean coordinates of control peaches and of all cold stored samples under SL (both after 0.5 and 5.5°C) were used.

The PLS model, based on the whole metabolic data set as predictor variable and cumulative temperature as response variable, reveals that the two low temperatures (0.5 and 5.5°C) only in part induced selective effects as samples from the two conditions cluster moderately overlapped, both considering peaches under storage and during shelf-life (**Figure 5A**). Almost 80% of the variability is explained by the first two factors of this model (about 65 and 15% for the first and second factor, respectively).

The most of the variability present in this dataset is explained by the first factor of this PLS model, along which samples are well positioned according to the employed continuous variable of the cumulative temperature. Peaches stored under different conditions indeed form a line representing their cumulative temperature history and, in general, peach ripening direction moves from the left toward the right quadrants (from blue to red colors) of the plot (**Figure 5A**). The red arrow drawn in the score plot of **Figure 5** represents the displacement relative to the overall variation existing between peaches under storage and SL condition. Its direction and length represent the shift of cold stored samples toward their metabolic phenotype during SL.

Employing VIP scores to select variables contributing the most to cluster separation, several metabolites such as malic, pyruvic, and threonic acid, glucose-6P, sorbitol, putrescine, most of the detected aldehydes [namely pentanal, 2-pentenal, hexanal, 2-hexenal, 2-hexenal(E), heptanal, 2-heptenal, 2-octenal, nonanal and decanal], several alcohols (such as hexanol, 2-hexen-1-ol, 2-ethylhexanol and octanol) and esters (namely hexyl and 2-hexenyl acetate) strongly correlate with samples at harvest (T0) and under cold storage (**Figure 5B**). All these metabolites sit on the two left quadrants of the loading plot and are mainly related to fruit at harvest and under 0.5°C storage, with these latter samples maintaining an unripe-like profile until the end of the trial. These metabolites also show some degree of correlation with samples under 5.5°C storage and fruit kept for 3 days under SL condition following 0.5°C storage (**Figure 5B**). Samples under 0.5°C are characterized by higher level of putrescine throughout the experimental period. On the other hand, peaches during storage at 5.5°C, as well as 0.5°C stored fruit after SL at 20°C, are richer in sorbitol.

Almost all control peaches and 5.5°C stored samples after a SL period sit on the right quadrants of the score plot in **Figure 5**. These two groups of samples represent peaches with the most advanced ripening phenotypes recorded in the trial. These fruit are highly associated with organic acids (chromogenic, citramalic, fumaric, gluconic, glucuronic, mucic, oleic and quinic acid), sugars (altrose, arabinose, fructose, fucose, glucose, maltose, melibiose, and xylose) and other molecules, such as alanine, β-alanine, fucitol, maltitol, and epicatechin. Among volatiles compounds, all the identified lactones, some esters, namely ethyl, isobutyl, 2-pentenyl, 4-pentenyl and 2-butoxyethyl acetate, and benzaldehyde are higher in these samples.

Considering compounds displayed in the two right quadrants of **Figure 5B**, it is worthy to note that marked differences are present when comparing control and cold stored +SL samples. In fact, while the top right quadrant contains compounds which are more related to control fruit, the molecules in the bottom right quadrant show greater amounts in peaches after storage at 0.5°C. Samples after 5.5°C storage are placed in between these two latter conditions showing intermediate compound levels (**Figure 5A**). All identified lactones, except for γ-hexalactone which is higher in control fruit, appear to be highly restored after 5.5°C but not after 0.5°C storage. Moreover, cold stored fruit show higher level of benzaldehyde, sugars, several organic acids and epicatechin. On the other hand, control fruit and 5.5°C stored peaches show higher amounts of esters, fumaric, glucuronic, citramalic and mucic acid, β-alanine and alanine (**Figures 5A,B**).

### Comparative Analysis of Post-harvest Ripening and Cold Storage-Induced Metabolic Changes

For comparative purposes and in order to better analyze the effects of cold storage on the fruit ripening metabolism, a heatmap has been produced considering the metabolic and aroma profiling during the post-harvest ripening (comparing T0 with 7 days later at room temperature) (Supplementary Figure S2). Sugar levels mirror the TSS content, with FL and RL showing the highest concentrations. Also sugar alcohols, especially maltitol and arabitol, are higher in FL and RL samples, respectively. Amino acids are generally found at the highest levels in RH, whereas organic acids are similar between cultivars during normal ripening, but RH and FL peaches show abundance of organic acids having high VIP score, thus contributing to cultivar clustering considering the whole experiment (**Figure 4**). The identified flavonols (catechin and epicatechin) are higher in FL and RL peaches, while putrescine and urea reach the highest level in RL and RH samples, respectively (**Figure 4** and Supplementary Figure S2).

Regarding VOCs, C6 compounds are generally higher in FL peaches, while alcohols appear similar in the cultivars studied, even though RH and RL show higher levels of several compounds belonging to this chemical class (pentanol, octanol, nonanol, 2-methylbutanol, isobutanol, 3-pentanol and 1-penten-3-ol). Aldehydes display a relatively homogeneous pattern of accumulation while esters are more variable, with RH and RL generally showing higher levels. Considering the whole experiment, γ-hexalactone and γ-octalactone are higher in RL while γ-decalactone and δ-octalactone show similar results for the three cultivars (**Figure 4** and Supplementary Figure S2).

**FIGURE 6:**
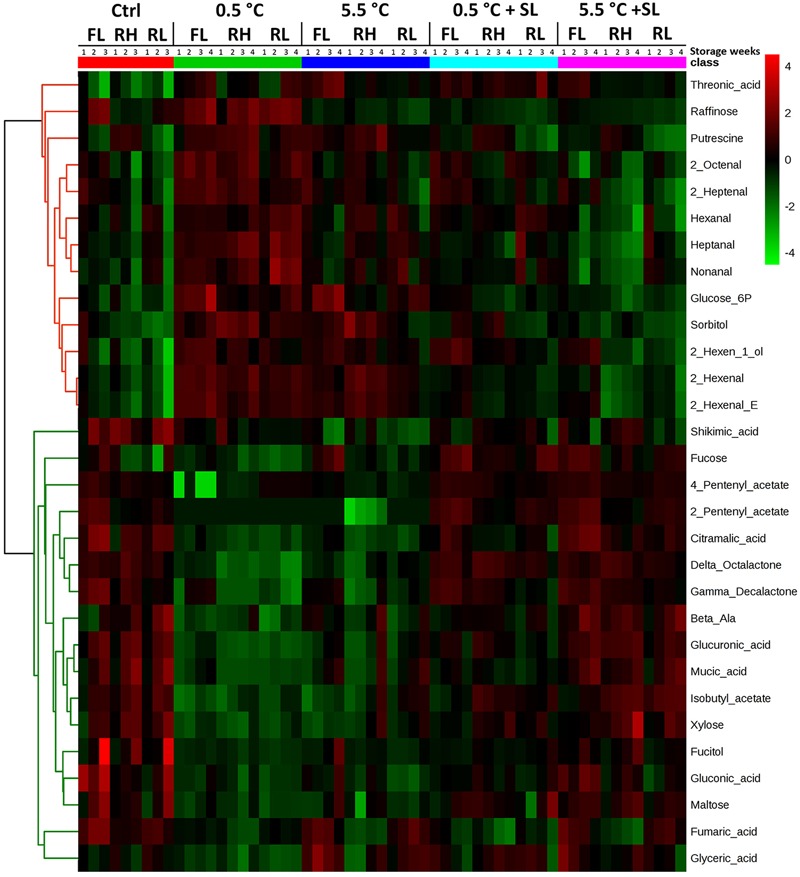
Heatmap analysis showing the fold change of top 30 compounds, sorted via ANOVA test (*p* ≤ 0.05), contributing to the separation of the different treatments (both at the end of cold storage and after shelf-life, SL). Samples from all the tested conditions have been included in the analysis. Each column representing the specific cultivar (‘Red Haven’, RH, ‘Regina di Londa’, RL, and ‘Flaminia’, FL) is divided in sub-columns, one for each storage week (up to 3 or 4 for control and cold stored samples, respectively): each cell represents the average of three biological replicates. The correlation coefficients employed in order to group the different features were calculated by applying Pearson correlation using average as clustering algorithm. In this analysis features have been auto-scaled (mean-centered and divided by the standard deviation of each variable) in order to better visualize differences between treatments. Relative level of each compound was normalized separately for each cultivar on its amount at harvest (T0) in order to get information on compound levels along time. The color scale from green (–4) to red (4) is proportional to the compounds amount, given as relative fold change. The fold change was calculated using the formula: FC = –log_2_[mean(T0)/mean (T1, T2, T3, or T4)], where T from 0 to 4 is the relative intensity of each compound after 0, 1, 2, 3, or 4 weeks of storage, with or without a SL period depending on the specific condition.

A second heatmap analysis has been performed on the whole dataset applying ANOVA to filter significant information in order to visualize the main results of the experiment. Considering the whole experimental plan, two main clusters can be identified in the heatmap reported in **Figure 6**. The top half of the graph (red cluster) reports compounds that in general show higher levels under cold storage (0.5 and 5.5°C) while lower values are detected after 3 days of SL when a recovery of ripening occurs [raffinose, putrescine, hexanal, 2-hexenal, 2-hexenal(E), heptanal, 2-heptenal, 2-octenal, nonanal, glucose-6P, sorbitol and 2-hexen-1-ol]. The second cluster spanning the bottom half of the heatmap (green cluster) includes instead metabolites that appear to be ripening-induced in control fruit (with the exception of fucose in RH), and decrease during storage at 0.5°C, but increase during SL after cold storage (β-alanine, fucose, maltose, xylose, fucitol, citramalic, fumaric, glucuronic, gluconic, glyceric, mucic and shikimic acids, isobutyl, 2-pentenyl and 4-pentenyl acetate, lactones). For a more complete overview on these results, detailed heatmap analyses are reported in Supplementary Figures (S3, S4, S5A,B).

Analyzing the two clusters reported in **Figure 6** more in detail, it appears that raffinose increases mainly at the lowest storage temperature (0.5°C), accumulating in all three varieties. Other compounds [2-hexenal, 2-hexenal(E), 2-hexen-1-ol, sorbitol] are more widely cold-related showing higher levels in both 0.5 and 5.5°C samples. Threonic and shikimic acids show opposite trends: in fact, threonic acid decreases in control samples of all cultivars and shows a general increase in cold samples (both before or after SL), while shikimic acid remains in general at low levels in cold stored peaches.

Considering compounds increasing during ripening, three separated sub-groups of metabolites are identified. The first group includes compounds such as citramalic acid, 2-pentenyl and 4-pentenyl acetate, δ-octalactone and γ-decalactone that show an increasing trend also during SL of cold stored fruit, both under 0.5 and 5.5°C. A second group includes metabolites (glucuronic and mucic acids, β-alanine, xylose, and isobutyl acetate) that increase in all cultivars during SL after 5.5°C storage, with more variable responses during SL after 0.5°C. The third sub-group identified in the heatmap is characterized by compounds (gluconic, glyceric and fumaric acids, maltose and fucitol) that increase during postharvest ripening but do not show any clear increasing or decreasing trend during SL after storage under both temperatures (**Figure 6**).

Considering some specific features of the three varieties, the accumulation of putrescine seems to be associated with RH ripening (also after cold storage), although a general increase of this compound is detected in 0.5°C-stored samples of all considered varieties. Another compound showing a similar behavior in RH fruit is sorbitol, which accumulates in this variety both under and after cold storage at 0.5 and 5.5°C, whereas it decreases in RH control peaches. Different from the other genotypes, RH samples seem to specifically respond to cold with a lower accumulation of fumaric acid in all the tested conditions (**Figure 6**). Moreover, considering cold storage, several other compounds, namely malic and shikimic acids, hexanal and 2-hexenal, enhance under 0.5°C in all varieties and decrease under 5.5°C only in FL and RL samples, while increase in RH cultivar (**Figure 6** and Supplementary Figure S3).

### Correlation Network: Metabolite-Metabolite Interactions and CIs-Related Metabolic and Aroma Profile Alterations

**FIGURE 7:**
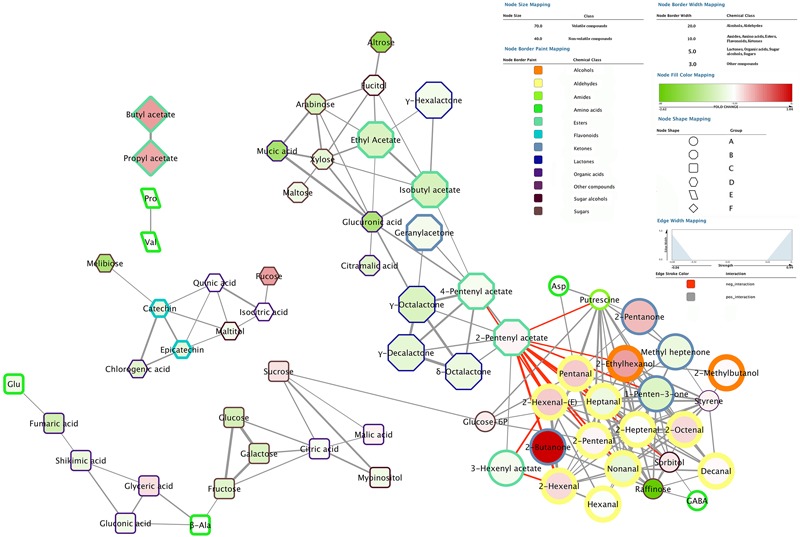
Correlation network analysis performed employing 0.7 as correlation coefficient cut-off. Nodes represent detected molecules showing significant positive or negative interaction between each other. Volatile compounds are reported with nodes of larger size compared to non-volatile nodes. Different node border color, from yellow to brown, and width are used to depict the different chemical classes. Nodes are filled with colors ranging from intense green to dark red which represent the fold change level of each compounds when comparing chilling injured (cold stored fruit, both low temperatures, +SL) vs. sound (control) fruit after 2 weeks of storage. Positive values (compounds more abundant in chilling injured peaches) are reported in red while negative values (compounds more abundant in sound peaches) are depicted in green. Different node shapes are used to depict different groups of compounds: circles, octagons, squares, hexagons, parallelograms, and diamonds represent group A, B, C, D, E, and F, respectively. Groups A, B, and C are linked together in the network while groups D, E, and F are separated from each other. Gray and red edges represent positive and negative interactions, respectively, whereas edge thickness is directly related with the strength of the interaction: the larger the thickness the stronger the interaction.

The correlation network analysis results in the identification of six different groups (A, B, C, D, E, and F) (**Figure 7**). Among them, group A, B, and C are linked together while group D, E, and F are separated.

The aldehyde class is the most evident cluster sitting in the bottom right corner of the figure (group A). In this mixed group also other chemical classes, which are positively correlated with aldehydes, are present. Among them, volatile compounds are represented by alcohols (2-ethylhexanol and 2-methylbutanol), several ketones (2-butanone, 2-pentanone, 1-penten-3-one and methyl heptenone) and styrene. On the other hand, primary metabolites which share with aldehyde group a positive interaction are putrescine, sorbitol, raffinose, glucose-6P and some amino acids (aspartate and GABA).

Two esters, namely 2-pentenyl and 4-pentenyl acetate, which are highly induced under SL condition, link group A to the second most important group present in the top right corner of the network (group B, **Figure 7**). These two compounds, together with 3-hexenyl acetate (which sits in group A), are negatively correlated with compounds in cluster A and positively correlated with all the other molecules present in group B. All the identified lactones sit in group B and, except for γ-hexalactone, are placed very close to each other also showing one of the highest level of correlation recorded (represented by the thickness of the edges). Also other esters, which are highly produced/accumulated under SL, namely ethyl and isobutyl acetate, are included in group B (**Figure 7**). This second large cluster (group B) also includes several organic acids, sugars and sugar alcohols. It is interesting to note that arabinose, xylose, glucuronic, and mucic acid, which are linked to cell wall metabolism, appear to be highly correlated.

An additional element of the network, which sits at the bottom of **Figure 7** (group C), is positively linked to aldehydes cluster via the connection between sucrose and glucose-6P. This latter sugar positively interacts with putrescine, pentanal, 2-pentenal and heptanal on one side, but also with sucrose on the opposite side, which in turn is positively connected with all sugars, organic acids, and amino acids present in group C. Among these metabolites, fructose, glucose, and galactose show the strongest interaction, followed by myoinositol and citric acid.

The top left side of the network includes three isolated groups (D, E, and F). The biggest cluster includes all the detected flavonols (epicatechin and catechin), which sit very close to each other and show positive interaction with several other primary metabolites, namely chlorogenic, quinic and isocitric acid, melibiose, fucose, and maltitol (group D). The other two groups include only two positively correlated molecules: proline and valine (group E), and butyl and propyl acetate (group F). Both couples have a specific trend, different from all other compounds reported in the network, from which they are completely separated (**Figure 7**).

In order to identify possible markers of physiological disorders and, at the same time, define their metabolic correlations, a specific analysis has been performed only considering fruit showing (cold stored fruit after SL) or not showing (control) CI symptoms after 2 weeks of storage. Indeed, at this sampling time the two conditions result in clearly different responses in terms of CI incidence (**Figure 2**). In **Figure 7** nodes filled with different intensity of red and green colors indicate the fold change level of each compound when comparing chilling injured vs. sound (control) peaches. Several compounds result to be more abundant in chilling injured peaches. Among them, 2-butanone shows the highest positive fold change, followed by butyl and propyl acetate, 2-ethylhexanol, 2-pentanone, several aldehydes [pentanal, 2-pentenal, 2-hexenal, 2-hexenal(E) and 2-octenal] and fucose. Other molecules have lower positive fold changes, such as styrene, sorbitol, glucose-6P, sucrose, malic, and glyceric acid (**Figure 7**), and are slightly more abundant in the injured fruit.

On the other hand, chilling injured peaches have lower levels of specific sugars, such as raffinose, altrose, and melibiose. Other sugars, namely maltose, arabinose, xylose, glucose, fructose, galactose, fucitol, myoinositol, and maltitol, follow the same trend but with a lower intensity (fold change) of decrease. Considering the other chemical classes, glucuronic and mucic acids decrease with the highest negative fold change, followed by fumaric, chlorogenic, citramalic, gluconic, citric, shikimic, and glutamic acids (**Figure 7**).

Flavonols, lactones as well as all the esters reported in the network, with the exception of 2-pentenyl acetate and some ketones (methyl heptenone and 1-penten-3-one), ketone-derivatives (geranylacetone), aldehydes (heptanal and nonanal) and proline show lower levels in injured fruit (**Figure 7**).

## Discussion

### The Main Metabolic Effects of Low Temperature Storage and the Incidence of CIs

Cold storage deeply affects the metabolism of harvested fruits including peaches. Recent papers reported changes in composition and metabolic pathways induced by low temperature in harvested peaches (Lauxmann et al., 2014; Bustamante et al., 2016, 2018; Yu et al., 2016, 2017). The data presented herein, result of an integrated metabolomics approach, provide additional information concerning different and common responses to refrigeration of three genotypes in terms of quality parameters, incidence of the main CIs affecting cold stored peaches, and selected metabolic changes, in fruit analyzed at the end of refrigerated storage and after SL.

It is well know that the responses to cold storage strongly depend on the genetic background: peach fruit storability varies when comparing early vs. late harvest cultivars, different phenotypes (white vs. yellow fleshed) or different physiological traits (melting vs. non-melting flesh). The current integrated metabolomics approach, which combines VOCs and metabolic profiling, points out that the different behavior in terms of loss of firmness and incidence of the studied CIs (flesh browning and bleeding, and mealiness) observed in RH peaches compared to RL and FL, is paralleled by a clear separation when multivariate analyses are performed (**Figures 3, 4**). Marked differences have been found comparing the hundreds of known cultivated peach varieties in terms of aroma, flavor, sweetness/acidity ratio, and other metabolic traits (Botton et al., 2006; Cai et al., 2015; Monti et al., 2016). This is confirmed in the present work concerning FL and RL genotypes that showed higher sugar, sugar alcohol and flavonol contents, while RH was the richest in terms of amino acids also displaying, together with FL, high organic acid levels. Similarly to other published results (Zerbini et al., 2011; Cano-Salazar et al., 2012; Wang et al., 2013), flesh firmness changed very little in all tested peach cultivars under the lowest temperature (0.5°C) throughout 4 weeks of storage. In addition to the positive effects on firmness, fruit stored at 0.5°C displayed a lower incidence of flesh browning and higher extractable juice as compared to 5.5°C samples with different behavior in the three tested varieties, in particular considering RH. This confirms that the onset and development of CIs largely depend on the genetic background (Pons et al., 2015; Bustamante et al., 2016). Tolerance or lower susceptibility to CIs is definitely a multigenic trait resulting in complex metabolic re-arrangements involving a number of metabolites. This means that it is unlikely that one single metabolite can be considered related to resistance or susceptibility. However, it is worthy to note that, compared to FL and RL, RH peaches showed unique patterns of accumulation of specific metabolites. The identification of specific compounds truly effective in alleviating CIs represents a key step toward practical applications of protocols aimed at optimizing cold storage of peach fruit.

Among these compounds, putrescine, a metabolite belonging to the class of polyamines, has been described in peaches as important compound effective in alleviating chilling injury (Cuevas et al., 2008; Lauxmann et al., 2014). Putrescine amounts were higher in 0.5°C samples, which showed lower levels of flesh browning but higher level of flesh bleeding, and increased more intensely in RH peaches, which displayed no flesh browning and very low levels of flesh bleeding, after cold storage (**Figures 2, 6**). RH was also characterized by the highest level of urea (Supplementary Figure S2), which has been associated with plant drought stress response and is induced by heat treatments, potentially contributing to reduce CI symptoms (Yancey et al., 1982; Lauxmann et al., 2014).

Sugar metabolism is also recognized to be profoundly affected by low temperature storage. Raffinose accumulation has been reported to be strongly induced in cold stored peaches (Lauxmann et al., 2014). It has been argued that this sugar could play an important role in CI tolerance, possibly acting as antioxidant or signal that mediates stress responses, but also playing a direct role stabilizing membrane system by entering the lipid head region (Korn et al., 2010; Davik et al., 2013). This sugar was found to be negatively correlated to mealiness symptoms and proposed as potential marker (Bustamante et al., 2016). Our results, showing a significant increase in raffinose under 0.5°C (**Figure 6**), support this hypothesis.

Xylose accumulation, one of the main constituents of cell wall hemicellulose, is induced after cold storage, especially in mealiness-susceptible genotypes possibly due to specific cell wall reconfiguration (Brummell et al., 2004; Fruk et al., 2014), and a potential implication of β-xylosidase in CI tolerance has been previously reported (Falara et al., 2011; Genero et al., 2016). All cultivars tested in the current work accumulated xylose after storage in particular when 5.5°C temperature was applied (**Figure 6**), as also reported by Bustamante et al. (2016). This sugar was not restored properly following 0.5°C, potentially contributing to keep higher extractable juice levels during SL after 0.5°C storage (**Figures 2, 6**). RH samples during SL after 5.5°C storage showed higher xylose levels (**Figure 6**), possibly linked to the lower values of extractable juice recorded after storage under this temperature (**Figure 2**).

Amino acids accumulation is reported to improve resistance to CIs in several species (Purvis, 1981; Zhao et al., 2009). Different papers report both positive and negligible effects regarding amino acid accumulation in peaches. Proline and GABA were found to be not associated with the improvement of CI tolerance by Bustamante et al. (2016), while other authors demonstrated that GABA significantly promotes it, enhancing antioxidant enzymes activity (Lauxmann et al., 2014; Shan et al., 2016). Cao et al. (2016) showed that the positive effects of melatonin and heat treatments regarding CI incidence were associated with increased proline and GABA levels in peach fruit. In the present work, proline increased in both RL and FL samples under cold storage, while RH samples revealed a decreasing trend of this amino acid both under and after cold storage (Supplementary Figure S5A). However, RH fruit showed the highest levels of all detected aminoacids, including proline and GABA suggesting that these metabolites might indeed have a role in delaying/reducing the onset of cold storage disorders such as flesh browning and bleeding (**Figure 4** and Supplementary Figure S2).

Correlation network analysis revealed that raffinose, putrescine and GABA, as well as aspartate, glucose-6P and sorbitol, cluster together in the same group of compounds showing significant positive interaction (**Figure 7**). The fact that all of them increase under cold storage, especially under 0.5°C, could partly explain their clustering. Moreover, while raffinose strongly decrease at 2 weeks with the onset of CIs, putrescine and GABA, as well as aspartate show similar values in sound and chilling injured fruit after 2 weeks of storage (**Figure 7**). This observation suggests that all these molecules are quickly metabolized during SL after cold storage with raffinose decreasing faster in relation to the onset of CIs. RH peaches showed the strongest putrescine increase under cold storage and, in general, the highest level of GABA (and aspartate), whereas raffinose had the same trend and levels in all the cultivars, possibly indicating no or limited role of this sugar in the onset/incidence of CIs in peach fruit.

### Metabolome and Volatilome: Genotype- and Temperature-Related Differences

The metabolic processes differentiating between the two low-temperature conditions (under and after storage) regard both non-volatile and volatile compounds. As a general comment considering the PLS analysis and the red arrow reported in **Figure 5** it is evident that a lower difference is present between peaches under and after cold storage in comparison to the difference detected between peaches at harvest and after ripening at 20°C.

Considering volatilome, several compounds increased more at 0.5°C: aldehydes and alcohols were in general correlated with such condition (**Figures 5B, 6**), although some genotype-related differences were present (discussed below). It is well known that aldehydes are linked to immature fruit and decline with ripening, when lactones and esters increase (Ortiz et al., 2010; Zhang et al., 2011; Supplementary Figure S2). Indeed, higher levels of aldehyde and C6 compounds have been detected at harvest and then they decreased during normal ripening. These lipid-derived compounds are negatively linked with lactones since both these volatile classes originate from the same fatty acid precursors (Schwab et al., 2008).

Considering the accumulation trends, it could be argued that in cold stored peaches the common increase in aldehydes and alcohols may reflect a higher amount of free radicals at cellular level under both low temperatures due to LOX activity. A specific behavior has been detected in RH peaches: in fact, hexanal, 2-hexenal, heptanal, 2-heptenal, 2-octenal, and nonanal increased also at 5.5°C (**Figure 6** and Supplementary Figure S3). This observation suggests that, despite the better performances in terms of storability shown by RH samples, in this cultivar free radicals, result of aldehyde production, increased with similar intensity at both low temperatures. Given the fact that no flesh browning has been detected in RH peaches, it could be argued that a higher antioxidant activity protects peaches of this cultivar from oxidative processes. Almost all detected aldehydes are positively correlated with raffinose, putrescine, and GABA but only few of them showed higher levels with the onset of CIs, namely pentanal, 2-hexenal, 2-hexenal-(E) and 2-octenal, whereas others, such as heptanal and nonanal, revealed the opposite trend (**Figure 7**). Moreover, 2-pentenal, hexanal, 2-heptenal and decanal levels were not affected by CI symptoms, revealing a specific modulation of aldehydes metabolism after cold storage also in relation to CIs onset. Indeed, the importance of the plasma membrane and the lipid metabolisms in relation to susceptibility/tolerance of peaches to cold storage stress has been recently described by Bustamante et al. (2018).

The accumulation of several VOCs, present at low level in control fruit and showing a decreasing trend during ripening (2-hexen-1-ol, hexyl acetate, 2-hexenyl acetate, 4-methyl-2-heptanone, methyl heptenone, 1-penten-3-one, and 2-pentanone), was highly induced under cold storage (**Figures 5B, 6** and Supplementary Figures S3, S5B). Also after storage, samples showed higher levels of several of these compounds, especially considering ketones that, in the correlation network analysis, cluster together with aldehydes and alcohols (**Figures 5B, 6, 7** and Supplementary Figures S4, S5B), further highlighting the role and impact of fatty acid metabolism in the responses to cold stress. Ketones seem to be differently involved in the onset of CIs, some of them (2-butanone and 2-pentanone) highly abundant in injured peaches, others (1-penten-3-one and methyl hepteone) at higher levels in sound fruit. Considering the identified ketones, little information is reported in literature regarding peach storage, and a non-homogeneous behavior has been observed in cold stored peaches depending on genotype (Cano-Salazar et al., 2013). This class of compounds was found to be induced in peach also by hyperbaric, controlled atmosphere, and UV treatments (Yang et al., 2009), and during strawberry freezing and thawing (Larsen and Poll, 1995). All these observations could explain the detected increase of ketones, especially under the lowest temperature. It must be considered that esters- and ketones-derived volatiles are produced from alcohols and aldehydes precursors and, in addition, enzyme activities are temperature-dependent driving to specific and selective volatile biosynthesis under 0.5 or 5.5°C.

Ripening-related VOCs, such as all detected lactones, several acetic acid derived esters, namely isobutyl, ethyl, 2-butoxyethyl, 2-pentenyl, and 4-pentenyl acetate, were found to be higher under 5.5°C than 0.5°C storage, probably due to the less pronounced effect of this condition on ripening evolution (**Figure 5B** and Supplementary Figure S5B). Other esters, such as hexyl and 2-hexenyl acetate, were higher under 0.5°C storage. Some VOCs, such as 2-pentenyl and 4-pentenyl acetate, showed a restored accumulation after cold storage in all cultivars and treatments, appearing less sensitive to cold storage (**Figure 6**). On the other hand, the level of some other compounds, such as isobutyl acetate, γ-hexalactone and γ-octalactone was completely restored during SL after 5.5°C but only in part following 0.5°C storage (**Figure 5B** and Supplementary Figure S5B). Isobutyl acetate is a ripening-related compound as observed in melon by Obando-Ulloa et al. (2009). All these molecules increased during normal or post-storage ripening and grouped in a specific part of the network in **Figure 7**, pointing out a strong positive interaction among them. Furthermore, with the exception of 2-pentenyl acetate, their level decreased in chilling injured fruit after 2 weeks of storage revealing an altered ripening process related to CIs. These results also indicate that 0.5°C storage strongly affects peach fruit ripening, delaying softening but also reducing the ripening-related production of volatile compounds during SL. In RH and RL peaches lactone level revealed a higher increase after cold storage than in control fruit (**Figure 6** and Supplementary Figure S5B). Contrasting results describing a decrease (Cano-Salazar et al., 2013) or negligible changes (Xi et al., 2012) in lactone production after cold storage are reported, but a general decrease of these compounds has been associated with the onset of CIs (Raffo et al., 2008; Zhang et al., 2011, 2012). Our results reveal the presence of a strong genotype effect on lactone accumulation after cold storage and also point out that a relationship exists between CIs and the reduction of compounds belonging to this chemical class (**Figure 7**).

Among different VOCs, RH peaches showed extremely higher level of linalool as compared to the other cultivars, especially during SL following 0.5°C storage (Supplementary Figures S2, S4, S5B). This terpene was recorded in peaches at harvest but under low temperatures it was rapidly degraded (Raffo et al., 2008), as confirmed by the current results (Supplementary Figure S5B). To the best of our knowledge no information is available on linalool content in peaches during SL after cold storage. It is worth noting that linalool levels doubled after freezing and thawing in strawberry fruit (Larsen and Poll, 1995).

Sugars and sugar alcohols are generally described as osmoprotectant compounds under stress conditions and their concentrations are highly variable in peach cultivars and tend to increase under cold temperatures (Wang et al., 2013; Yu et al., 2016, 2017), as also confirmed by the current results (**Figures 4–6** and Supplementary Figures S2, S5A). RL and FL peaches, which showed CIs symptoms, displayed the highest amount of sugars and sugar alcohols (**Figure 4B** and Supplementary Figure S2). On the other hand, some specific compounds, such as sucrose and sorbitol, were found to increase more in RH than in the other samples during and after cold storage (**Figure 6** and Supplementary Figures S3, S4, S5A), and in particular during the 1st week of storage. Taking also into account the results of the correlation network analysis concerning sugars and sugar alcohols, it seems clear that the genotype-related modulation of sugar metabolism may represent a key factor in the response of peach fruit to postharvest cold stress conditions.

Shikimic and malic acids, the first employed as VOCs precursor during ripening and the latter generally decreasing during the same process, accumulated in all tested varieties under 0.5°C, with RH peaches showing increases of both acids also under 5.5°C, indicating a delay of ripening (**Figures 5, 6** and Supplementary Figures S3, S4, S5A). NADP-dependent isocitrate dehydrogenase enzyme, which catalyzes the conversion of malic acid in pyruvate, has been found to decrease in peach fruit stored at 0°C (Zhang et al., 2010) thus confirming the current results concerning malic acid. Both shikimic and malic acids sit in the same group (C) of the network in **Figure 7**, with the former positively interacting with fumaric, gluconic and glyceric acids and the latter with citric acid and sucrose.

Glucuronic and mucic acids showed higher levels under 5.5°C than 0.5°C (**Figures 5, 6** and Supplementary Figures S3, S5A) and this may be related to a faster loss of flesh firmness. A relationship between glucuronic and mucic acid and softening (pectin metabolism) has been reported by Prasanna et al. (2007), Bar-Peled and O’Neill (2011), and Lee et al. (2012). After cold storage at 0.5°C glucuronic and mucic acids are not restored properly and this might be related to the reduced loss of firmness observed in these samples. These molecules cluster together with other ripening-related compounds in the correlation network (group B) and seem to be affected also by physiological disorders, decreasing in chilling injured fruit. All compounds belonging to group B, except for 2-pentenyl acetate, decreased in relation to the onset of CIs (**Figure 7**). This is also the case of citramalic acid, a metabolite that has been associated with responses to cold storage (and high carbon dioxide treatments) in apples (Fernández-Trujillo et al., 2001).

Finally, the observed increase in glyceric acid in peaches stored at 5.5°C, in particular in samples affected by CIs might be related to the presence of oxidative stress conditions and may affect glycolytic activity and amino acids accumulation (Verbruggen and Hermans, 2008; Obata and Fernie, 2012).

## Conclusion

Despite the marked genotype-related diversity observed, some conserved metabolic changes in response to cold stress, both during cold storage and after subsequent SL, have been observed. All cultivars revealed a pronounced increase in raffinose, glucose-6P, fucose, xylose, sorbitol, GABA, epicatechin, catechin, and putrescine, flanked by a decrease in citramalic, glucuronic, mucic, and shikimic acids under cold storage. Also aroma compound profiles revealed common changes in response to cold storage: as an example, aldehydes and alcohols generally accumulated more during cold storage while esters and lactones production was strongly inhibited. During SL after refrigerated storage, more specific responses were detected, with each variety behaving in a specific way, even though some common responses, such as the increased production of ethyl acetate, were detected.

The physiological responses involved in the lower incidence of flesh browning and bleeding in RH compared to RH and FL appear to be related to compounds with antioxidant activity and metabolites that act as osmotic protectants and membrane stabilizers. Based on these results, higher levels of compounds such as urea and amino acids, together with a pronounced increase in putrescine, sorbitol, maltitol, myoinositol, and sucrose concentrations are positively associated with a reduced susceptibility to CIs. Our integrated metabolomics approach clearly points out that symptoms and metabolic mechanisms related to mealiness, flesh bleeding, and browning in cold stored peaches should be evaluated separately, since it seems unlikely that one single metabolite can be considered related to resistance or susceptibility.

## Author Contributions

PT and SB designed the experimental trials. SB and RT performed the experiments, collected the samples, and performed the metabolic analyses. SB, MH, BN, and PT analyzed the data and discussed the results. SB, MH, and PT wrote the article. All authors read and approved the final manuscript.

## Conflict of Interest Statement

The authors declare that the research was conducted in the absence of any commercial or financial relationships that could be construed as a potential conflict of interest.

## Footnotes

1 http://www.baderlab.org/Software/ExpressionCorrelation

2 www.cytoscape.org
