# Supplementary material for: Metabolic Responses to Low Temperature of Three Peach Fruit Cultivars Differently Sensitive to Cold Storage
Source: Front Plant Sci. 2018 May 28;9:706. doi: 10.3389/fpls.2018.00706 (PMC5985494; doi:10.3389/fpls.2018.00706)
Supplement: Supplementary file 1 [file Data_Sheet_1.PDF]

Supplementary material - Metabolic responses to low temperature of three peach fruit cultivars differently sensitive to cold storage

Analytical technique	Compound	RT	RI	QI	CAS number
Derivatization GC-MS	Pyruvic acid **A**	7,92	1063	174	99124-30-8
	Lactic acid **C**	8,14	1076	147	50-21-5
	Oxaloacetic acid **A B C**	8,30	1086	333	328-42-7
	Alanine **A B C**	8,77	1116	116	56-41-7
	Valine **A B C**	10,40	1225	144	72-18-4
	Urea **A B C**	10,69	1246	147	57-13-6
	Glycolic acid **A C**	10,79	1253	147	79-14-1
	Phosphoric acid	11,26	1286	299	7664-38-2
	Threonine	11,41	1296	117	72-19-5
	Proline **A B C**	11,54	1306	216	37159-97-0
	Maleic acid **A B C**	11,62	1312	147	110-16-7
	Glycine **A B C**	11,71	1318	102	56-40-6
	Succinic acid	11,93	1334	147	110-15-6
	Glyceric acid **A B**	12,09	1346	147	473-81-4
	Fumaric acid **A C**	12,25	1358	245	110-17-8
	Serine **A B C**	12,45	1373	204	56-45-1
	β-Alanine **A C**	13,27	1437	174	107-95-9
	Citramalic acid **A C**	13,94	1491	247	597-44-4
	Malic acid **A**	14,21	1514	147	617-48-1
	Meso-erythritol **A B**	14,41	1531	103	149-32-6
	Aspartic acid **A B C**	14,59	1545	232	56-84-8
	GABA	14,73	1553	57	56-12-2
	Threonic acid **A B C**	15,07	1584	292	7306-96-9
	Asparagine **A B C**	15,31	1604	147	70-47-3
	Glutamic acid **A B C**	15,67	1636	156	25513-46-6
	Xylose **A B**	16,13	1677	103	609-06-3
	Arabinose	16,48	1709	217	147-81-9
	Fucose **A C**	16,63	1723	204	6696-41-9
	Arabitol **A C**	16,84	1743	103	488-82-4
	Putrescine	17,02	1760	103	110-60-1
	Fucitol	17,46	1802	147	13074-06-1
	Shikimic acid **A B**	17,79	1833	204	138-59-0
	Citric acid **A B C**	17,89	1843	273	77-92-9
	Isocitric acid **A B C**	17,98	1850	273	320-77-4
	2-Keto-Gulonic acid **A B C**	18,03	1856	147	342385-52-8
	Altrose **A**	18,29	1881	204	1990-29-0
	Quinic acid **A B C**	18,47	1898	345	77-95-2
	Fructose **A C**	18,53	1904	147	57-48-7
	Glucose **A C**	19,02	1954	319	2280-44-6
	Sorbitol **A B C**	19,34	1987	319	50-70-4
	Glucuronic acid	19,51	2001	333	528-16-5
	Gluconic acid **A B C**	20,01	2013	147	133-42-6
	Mucic acid	20,31	2020	333	526-99-8
	Myo-inositol **B C**	20,68	2028	217	87-89-8
	Galactose **A C**	20,95	2035	319	10257-28-0
	Oleic acid **A B C**	21,49	2047	117	112-80-1
	Stearic acid **B C**	21,72	2053	341	57-11-4
	Glucose-6P **C**	22,80	2078	147	299-31-0
	Sucrose **B C**	25,67	2561	361	57-50-1
	Maltose **A C**	26,14	2673	361	69-79-4
	Epicatechin **A C**	26,66	2799	368	490-46-0
	Catechin **A B C**	26,81	2867	368	154-23-4
	Melibiose **A B C**	27,03	2960	204	585-99-9
	Maltitol **A B C**	27,13	2872	204	585-88-6
	Chlorogenic acid **A C**	28,89	3775	307	202650-88-2
	Raffinose **B C**	30,43	4447	361	512-69-6
	Melezitose **A C**	30,59	4518	147	597-12-6
A = FL vs RH significantly different<br>B = FL vs RL significantly different<br>C = RL vs RH significantly different					

Table S1 - Metabolites detected via derivatization of the methanolic extracts of the samples analysed with GC-MS. The trimethylsilylated version of the compounds reported in the table have been identified after derivatization of peach samples. The CAS number listed here are referred to the non trimethylsilylated molecules. Bold letters indicate compounds that were found to be significantly different comparing via t-test analysis ($p \leq 0.05$) the three cultivars: 'A', 'B' and 'C' refer to FL vs RH, FL vs RL and RL vs RH comparison, respectively. RT: retention time (min); RI: retention (Kovats) index, HP-5MS capillary column; QI: quantification ion (m/z).

Analytical technique	Compound	RT	RI	QI	CAS number
HS-SPME-GC-MS	2-Butanone	1,96	579	43	78-93-3
	Ethyl acetate	2,10	601	43	141-78-6
	Isobutanol ⊙ **A**	2,17	613	55	78-83-1
	Isovaleraldehyde ⊙	2,32	637	58	590-86-3
	Butanol **A**	2,43	654	31	71-36-3
	1-Penten-3-ol ◆ **C**	2,55	674	57	616-25-1
	1-Penten-3-one ◆ **C**	2,60	681	55	1629-58-9
	2-Pentanone **A B C**	2,65	688	43	107-87-9
	Pentanal **A B C**	2,74	703	44	110-62-3
	3-Pentanol ◆ **C**	2,87	710	59	584-02-1
	Propyl acetate **A B C**	2,96	716	43	109-60-4
	2-Methylbutanol **A B C**	3,20	732	57	137-32-6
	4-methyl-2-heptanone **A B**	3,35	742	43	6137-06-0
	2-Pentenal ◆ **C**	3,48	750	55	1576-87-0
	Pentanol ° **B**	3,60	758	55	71-41-0
	Isobutyl acetate **A B**	3,77	770	43	110-19-0
	3-Hexanone [RH]	4,06	788	43	589-38-8
	Hexanal **A B**	4,24	801	56	66-25-1
	Butyl acetate **A B C**	4,55	813	43	123-86-4
	2-Hexenal-(E) **C**	5,26	842	83	505-57-7
	2-Hexenal **C**	5,46	850	41	6728-26-3
	3-Hexen-1-ol **A C**	5,59	856	67	928-97-2
	2-Hexen-1-ol **A B**	5,78	864	57	928-95-0
	Hexanol **C**	5,89	868	56	111-27-3
	Isoamyl acetate **A B**	6,06	875	43	123-92-2
	4-Pentenyl acetate **A B**	6,30	885	68	1576-85-8
	Styrene **A B C**	6,41	889	78	9003-53-6
	Heptanal **A B**	6,73	902	55	111-71-7
	Pentyl acetate **A B C**	7,12	914	43	628-63-7
	2-Pentenyl acetate **A B**	7,38	922	43	42125-10-0
	Butyl isobutyrate **B C**	8,09	945	57	97-87-0
	2-Heptenal **A B C**	8,41	955	41	2463-63-0
	Benzaldehyde **A B**	8,58	960	106	100-52-7
	Methyl heptenone **A B**	9,42	986	43	110-93-0
	3-Hexenyl acetate **A B**	10,10	1007	82	3681-71-8
	Hexyl acetate **A B**	10,32	1013	43	142-92-7
	2-Hexenyl acetate **A B C**	10,46	1017	43	2497-18-9
	2-Ethylhexanol **A B**	10,83	1028	56	104-76-7
	Benzeneacetaldehyde ⊙ **B**	11,32	1042	91	22-78-1
	γ-Hexalactone **A B C**	11,67	1052	85	695-06-7
	2-Octenal **A B**	11,83	1056	41	2363-89-5
	Octanol **B C**	12,30	1070	56	111-87-5
	2-Butoxyethyl acetate [RL]	13,07	1092	121	11-07-2
	Linalool **A B C**	13,32	1099	98	78-70-6
	Nonanal **B C**	13,56	1106	70	124-19-6
	Benzyl acetate [RL]	15,51	1161	43	140-11-4
	Nonanol **B C**	15,85	1171	85	143-08-8
	Decanal **A B**	17,04	1205	99	112-31-2
	γ-Octalactone **A B C**	18,81	1257	43	104-50-7
	δ-Octalactone **A**	20,45	1306	85	698-76-0
	Hexyl hexanoate [RH]	22,99	1384	256	6378-65-0
	Geranylacetone **A B C**	25,15	1453	117	689-67-8
	γ-Decalactone **A B**	25,61	1468	108	706-14-9
	Isopropyl palmitate	31,53	2001	43	142-91-6

[FL] = Identified only in FL; ◆ = Not detected in FL; **A** = FL vs RH significantly different
[RH] = Identified only in RH; ° = Not detected in RH; **B** = FL vs RL significantly different
[RL] = Identified only in RL; ⊙ = Not detected in RL; **C** = RL vs RH significantly different

Table S2 - Metabolites detected using HS-SPME-GC-MS technique. Letters indicate compounds that were found to be significantly different comparing via t-test analysis ($p \leq 0.05$) the three cultivars: ‘A’, ‘B’ and ‘C’ refer to FL vs RH, FL vs RL and RL vs RH comparison, respectively. The initials of each specific variety are employed to indicate compounds that were detected only in one of them ([FL], [RH] and [RL]), whereas ‘u’, ‘¢’ or ‘¤’ symbols are reported when a compound was not detected in FL, RH or RL cultivar respectively. RT: retention time (min); RI: retention (Kovats) index, HP-5MS capillary column; QI: quantification ion (m/z).

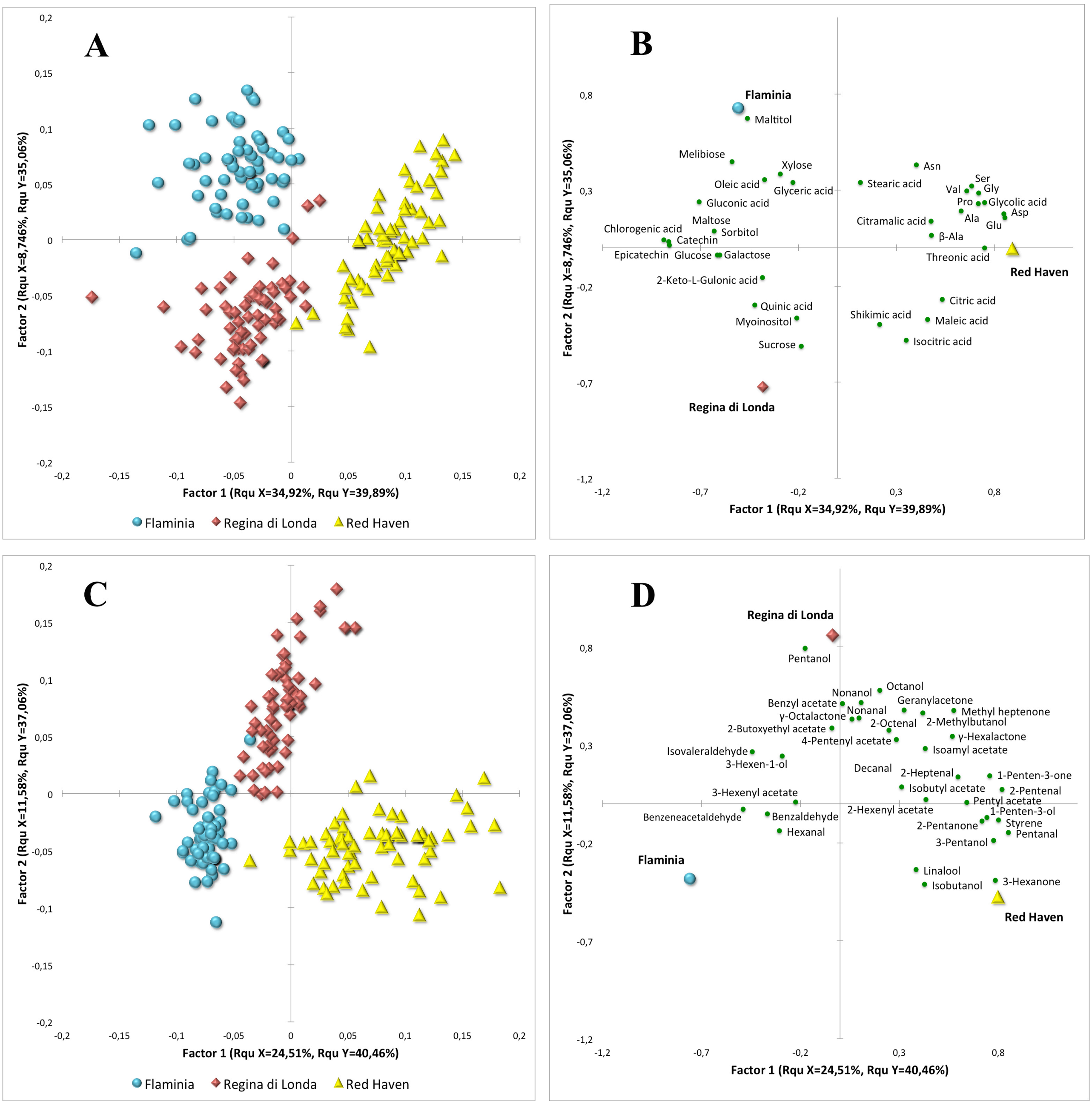

Figure S1 - Partial least squares discriminant analysis (PLS-DA). Results from both cold storage and shelf-life experiments are considered together. In the two PLS models cultivar has been employed as response variable while the identified compounds were used as predictor variables. These PLS-DA analyses have been performed using two different datasets including only metabolomics (**A/B**) or aroma profiling (**C/D**) results. Light blue circles, red diamonds and yellow triangles represent FL, RL and RH samples respectively.

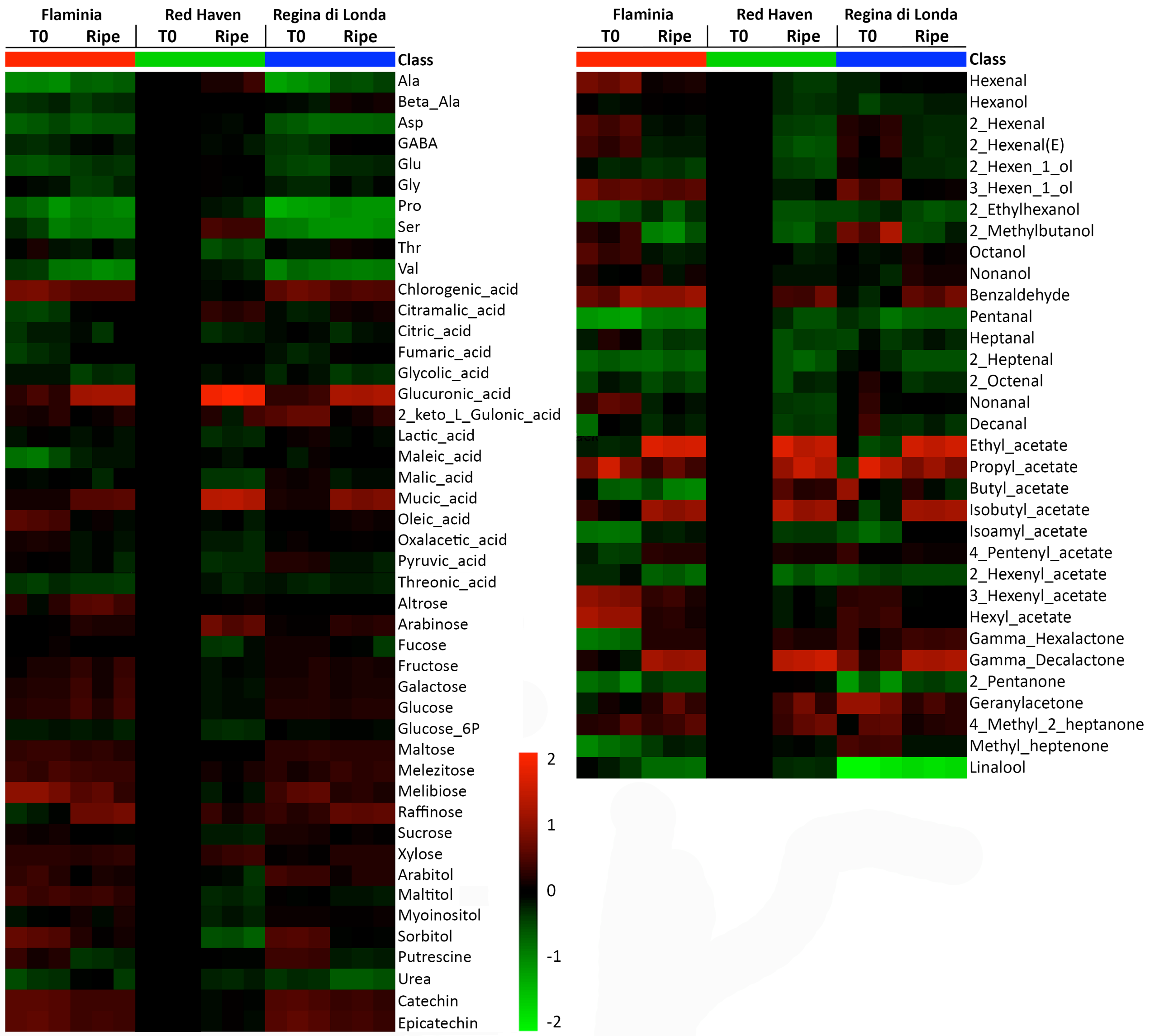

Figure S2 - Heat-map analysis showing compound trend accumulation during ripening in the three varieties. Only compounds that were in common to all cultivars (‘Red Haven’, RH, ‘Regina di Londa’, RL, and ‘Flaminia’, FL) and that showed important differences between them have been considered. Samples collected at harvest (T0) and after 7 days of post-harvest ripening at room temperature (Ripe), were analysed. Each T0 or Ripe column is divided in sub-columns that represent the three employed biological replicates. Relative level of each compound was normalized in relation to ‘Red Haven’ samples at T0 stage. The colour scale from green (-2) to red (2) is proportional to compounds amount, given as relative fold change. The fold change was calculated using the formula FC = $-\log_2$[mean (RH-T0) / mean (FL- or RL-T0)] and FC = $-\log_2$[mean(RH-T0) / mean (RH-, FL- or RL-Ripe)] to gain the value of FL- or RL-T0 and RH-, FL- or RL-Ripe cells respectively.

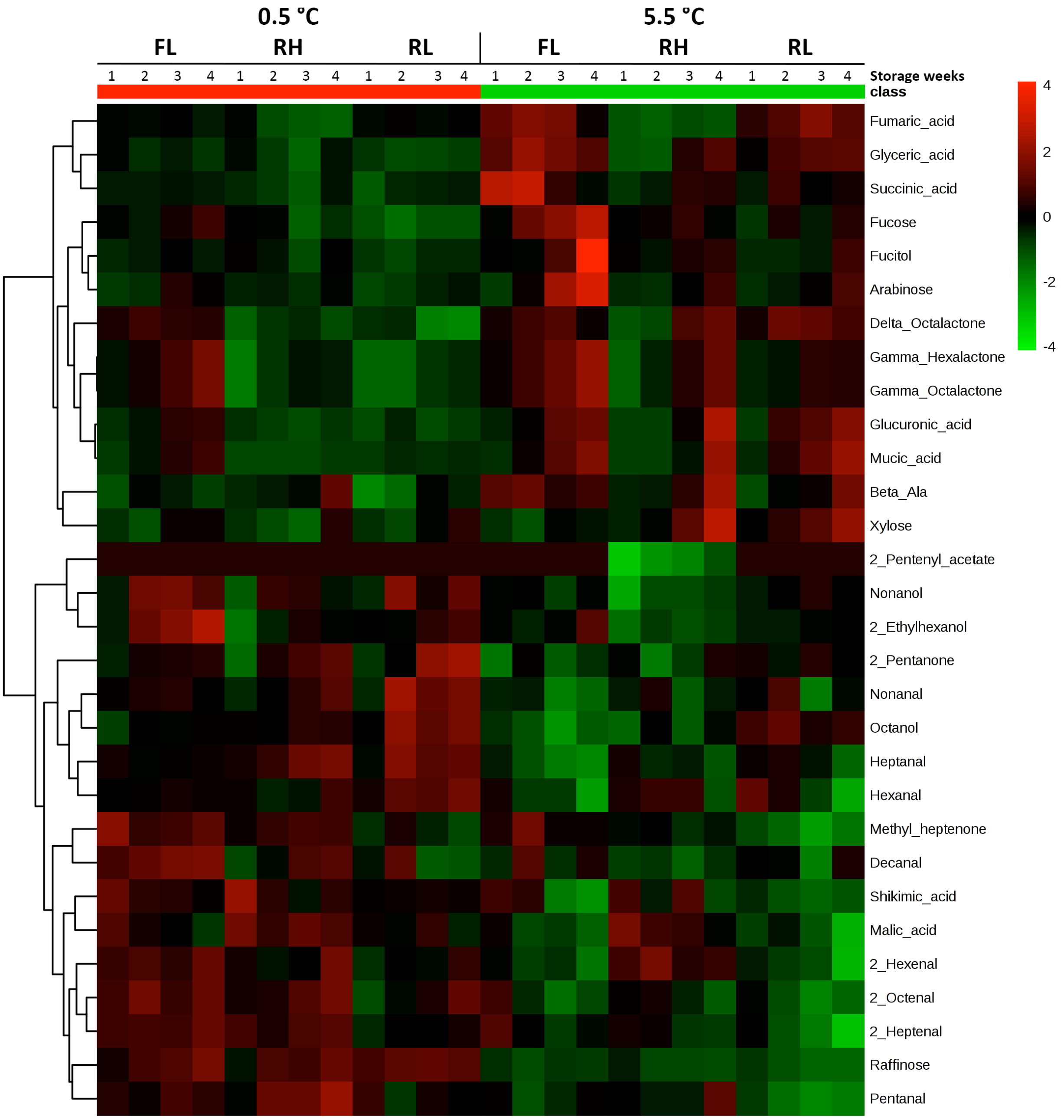

Figure S3 - Cold storage-induced modifications of peach fruit metabolome. Heat-map analysis showing the top 30 compounds, sorted via t-test ($p \leq 0.05$), contributing to the separation of samples stored under 0.5 and 5.5 °C. Only samples stored under the two low temperatures and immediately analysed were included in this analysis. The correlation coefficients employed in order to group the different features were calculated by applying Pearson correlation using average as clustering algorithm. In this analysis features have been auto-scaled (mean-centered and divided by the standard deviation of each variable) in order to better visualize differences between treatments. Relative level of each compound was normalized separately for each cultivar on its amount at harvest in order to get information on compounds trends along time. The colour scale from green (-4) to red (4) is proportional to compounds amount, given as relative fold change. The fold change was calculated using the formula: FC = $-\log_2$[mean(T0) / mean (T1, T2, T3 or T4)], where T from 0 to 4 is the relative intensity of each compound after 0, 1, 2, 3 or 4 weeks of storage.

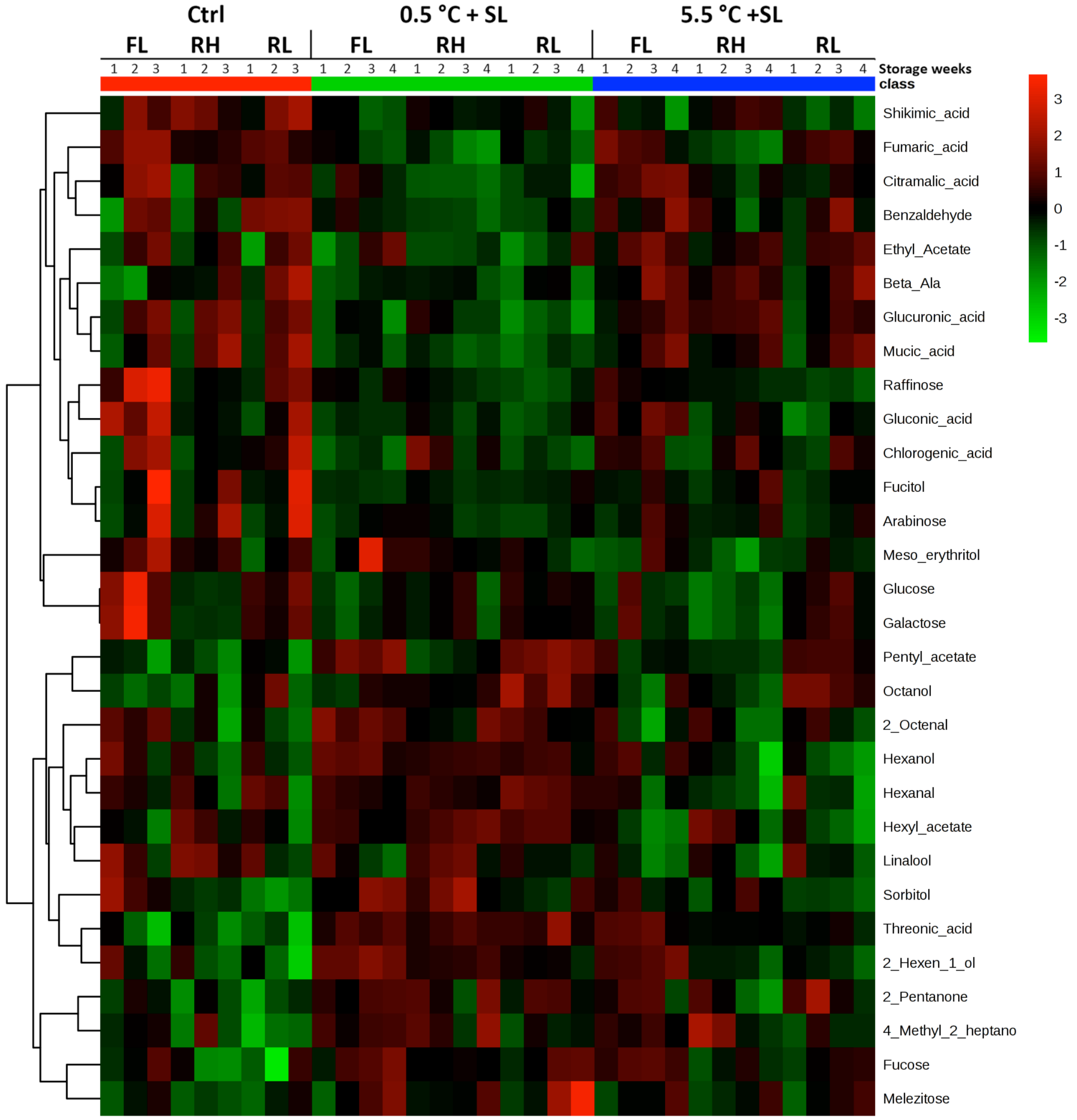

Figure S4 - The metabolic changes in peaches during shelf life at 20°C after cold storage. Heat-map analysis showing the top 30 compounds, sorted via ANOVA ($p \leq 0.05$; Fisher's least significant difference (LSD)), contributing to the separation of control treatment, kept at 20 °C, from samples that, after a period of cold storage (1, 2, 3 or 4 weeks), were kept for three additional days under SL conditions. Only samples undergone to a SL period are considered in the analysis. The correlation coefficients employed in order to group the different features were calculated by applying Pearson correlation using average as clustering algorithm. In this analysis features have been auto-scaled (mean-centered and divided by the standard deviation of each variable) in order to better visualize differences between treatments. Relative level of each compound was normalized separately for each cultivar on its amount at harvest in order to get information also on compounds trends along time. The colour scale from green to red is proportional to compounds amount, given as relative fold change. The fold change was calculated using the formula: FC = -$\log_2$[mean(T0) / mean (T1, T2, T3 or T4)], where T from 0 to 4 is the relative intensity of each compound after 0, 1, 2, 3 or 4 weeks of storage plus three days of shelf-life.

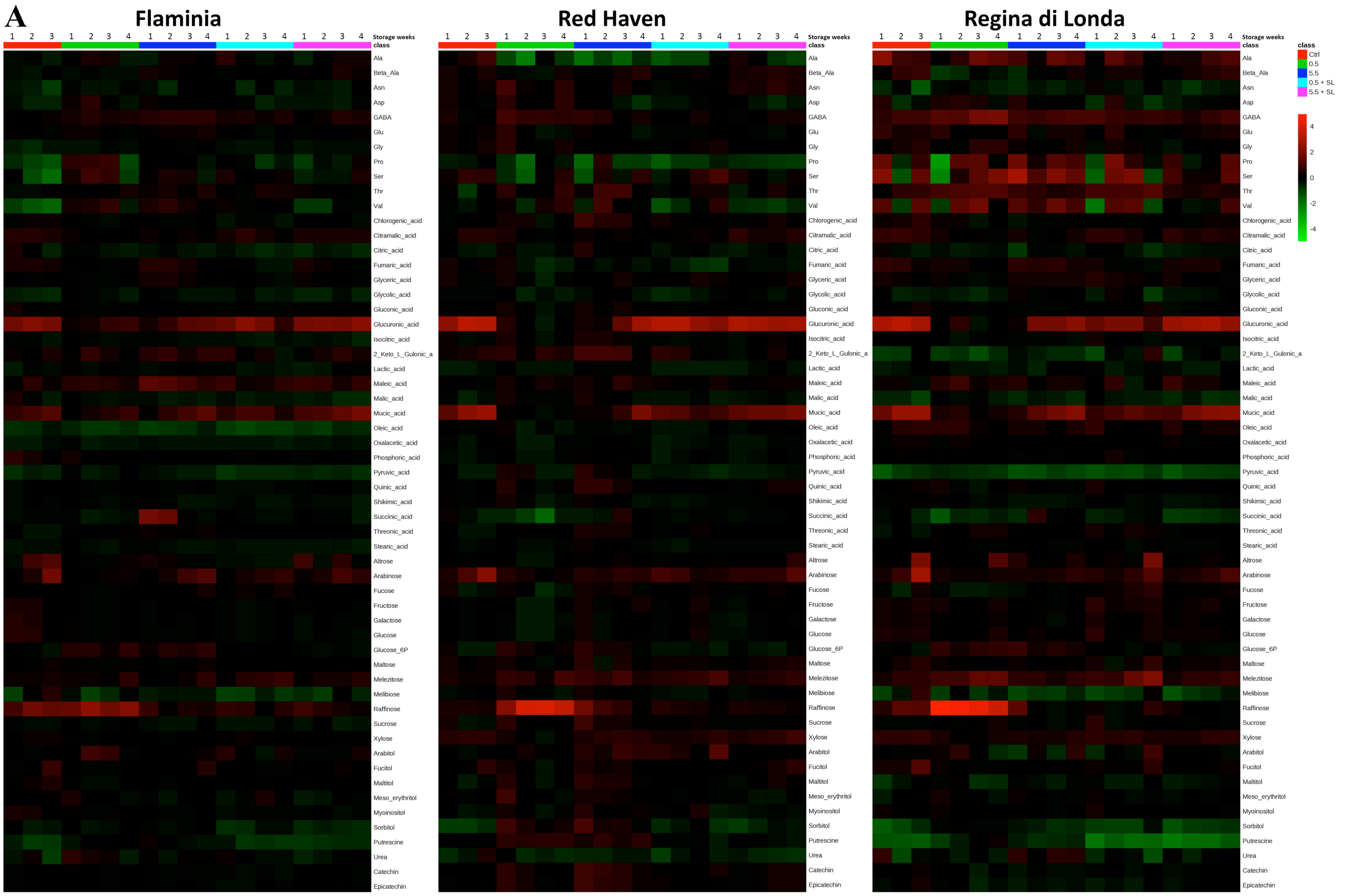
A
Flaminia
Red Haven
Regina di Londa
Storage weeks
class
1 2 3 1 2 3 4 1 2 3 4 1 2 3 4 1 2 3 4
Ala
Beta_Ala
Asn
Asp
GABA
Glu
Gly
Pro
Ser
Thr
Val
Chlorogenic_acid
Citramalic_acid
Citric_acid
Fumaric_acid
Glyceric_acid
Glycolic_acid
Gluconic_acid
Glucuronic_acid
Isocitric_acid
2_Keto_L_Gulonic_a
Lactic_acid
Maleic_acid
Malic_acid
Mucic_acid
Oleic_acid
Oxalacetic_acid
Phosphoric_acid
Pyruvic_acid
Quinic_acid
Shikimic_acid
Succinic_acid
Threonic_acid
Stearic_acid
Altrose
Arabinose
Fucose
Fructose
Galactose
Glucose
Glucose_6P
Maltose
Melezitose
Melibiose
Raffinose
Sucrose
Xylose
Arabitol
Fucitol
Maltitol
Meso_erythritol
Myoinositol
Sorbitol
Putrescine
Urea
Catechin
Epicatechin
class
Ctrl
0.5
5.5
0.5 + SL
5.5 + SL
4
2
0
-2
-4

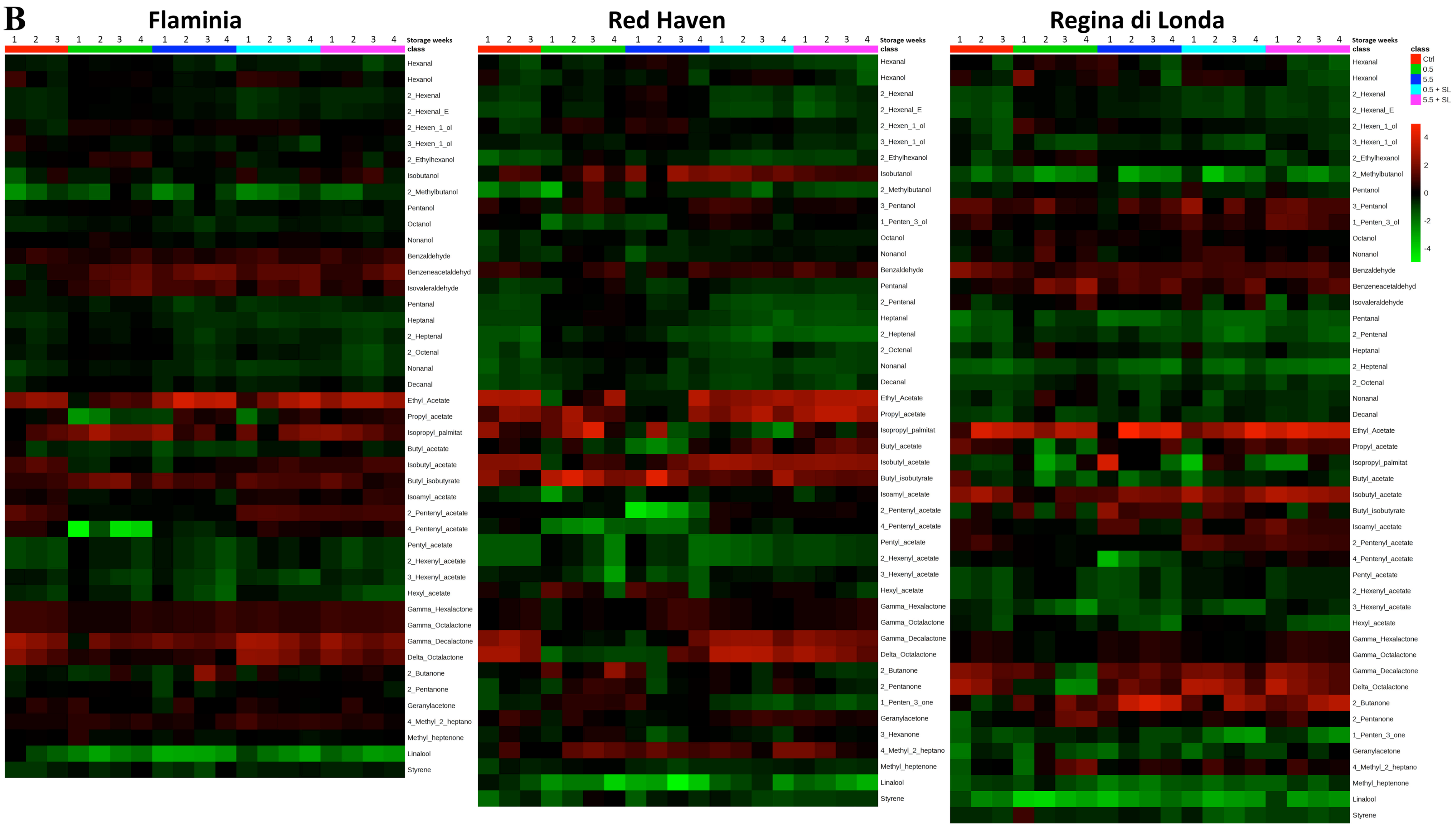
B
Flaminia
Red Haven
Regina di Londa
Storage weeks
class
Ctrl
0.5
5.5
0.5 + SL
5.5 + SL

Figure S5 - Cold storage and SL experiment considered together: A. Metabolomics dataset. **B.** Aroma profiling dataset. Both heat-map analysis A and B include samples from all the tested conditions. In this analysis features have been auto-scaled (mean-centered and divided by the standard deviation of each variable) in order to better visualize differences between treatments. Relative level of each compound was normalized separately for each cultivar on its amount at harvest in order to get information on compounds trend along time. The colour scale from green (-4) to red (4) is proportional to compounds amount, given as relative fold change. The fold change was calculated using the formula: FC = $-\log_2$[mean(T0) / mean (T1, T2, T3 or T4)], where T from 0 to 4 is the relative intensity of each compound after 0, 1, 2, 3 or 4 weeks of storage, with or without a shelf-life period depending by the specific condition.
